# Analysis of Subsurface Damage Based on K9 Glass Grinding

**DOI:** 10.3390/ma18194558

**Published:** 2025-09-30

**Authors:** Yao Liu, Jingjing Xie, Ruiliang Li, Jiankun Gao, Ming Li, Lin Sun

**Affiliations:** 1 Qinchuan Machine Tool & Tool Group Corp., Baoji 721009, China; lyxaut@163.com (Y.L.); lirl@qinchuan.com (R.L.); lishimin196@gmail.com (M.L.); 2 State Key Laboratory for Manufacturing Systems Engineering & School of Mechanical Engineering, Xi’an Jiaotong University, Xi’an 710049, China; 19853850611@163.com (J.G.); sunlin@xjtu.edu.cn (L.S.)

**Keywords:** K9 glass, grinding process, surface/subsurface damage

## Abstract

During the grinding process of K9 glass, various forms of surface damage—such as indentations and pitting—as well as subsurface damage—including cracks and residual stress—are generated. This paper focuses on the planetary grinding method utilizing bonded abrasives for both process research and subsurface damage detection. It examines the timeliness of grinding duration and analyzes the effects of abrasive grain size and grinding pressure on surface quality. Building upon the principle of differential etching, an improved HF chemical etching method is proposed to establish a relationship model that correlates the depth of subsurface damage with abrasive grain size, applied pressure, and surface roughness.

## 1. Introduction

**Figure 1:**
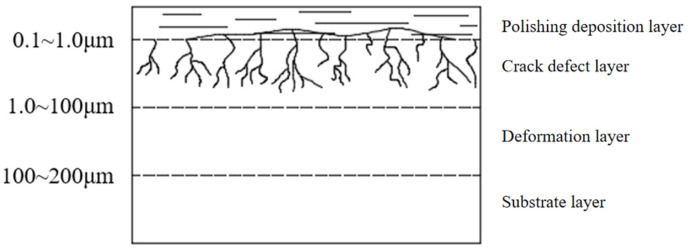
Subsurface damage structure diagram.

Optical glass inevitably develops subsurface defects [1], such as scratches, following grinding, lapping, and polishing processes due to external forces and its inherently brittle nature. Subsurface damage (SSD) encompasses cracks, scratches, and indentations present in both the surface and subsurface layers [2]. As illustrated in Figure 1, SSD is primarily distributed across three distinct layers: the polishing deposition layer (which enhances surface quality), the crack defect layer (resulting from grinding and lapping operations), and the deformation layer (induced by residual strains from abrasive cutting). These defects significantly impact the performance of optical components [3]. Therefore, the lapping process is crucial for eliminating surface damage and preparing for subsequent polishing. It uses HF etching [4] to analyze the detection of surface and subsurface damage depth and investigates the relationship between subsurface damage (SSD) and surface roughness (SR).

Choi JY et al. from Pusan National University [5] conducted lapping and polishing of D2 die steel using bonded abrasives, preparing the lapping and polishing discs through UV curing. The surface roughness of the workpiece after lapping and polishing was found to be satisfactory. Comparative analysis revealed that the material removal rate was 30% higher than that achieved with free abrasive processing. Huang WH et al. from the University of Arizona [6] employed Al_2_O_3_ bonded abrasives for lapping and polishing electroplated Cu films, resulting in an improved material removal rate. Lv BH et al. from Zhejiang University of Technology [7] performed experiments on silicon nitride ceramic balls utilizing both grinding methods, discovering that the material removal rate for bonded abrasive grinding was 20 times greater than that for free abrasive grinding. This finding underscores the high efficiency of bonded abrasive grinding and its significant potential for development. Li Biao from Nanjing University of Aeronautics and Astronautics [8] utilized both free abrasive grinding and bonded abrasive grinding techniques to process K9 glass, subsequently employing a magnetic fluid polishing spot method to assess subsurface damage depth in the processed samples. It was observed that the subsurface damage depth resulting from bonded abrasive grinding was less than that produced by free abrasive grinding, leading to superior surface quality post-processing.

Based on the solid abrasive grinding process, researchers have conducted extensive studies and improvements on solid abrasive grinding discs. Y. J. Ang et al. [9] concentrated on the material removal mechanisms in solid abrasive grinding, performing both theoretical analyses and experimental investigations into the wear characteristics of grinding discs as well as the force and motion dynamics of workpieces. Zhao P et al. from Zhejiang University of Technology [10] developed semi-fixed grinding discs derived from solid abrasive technology specifically for advanced ceramics. Their experiments with silicon wafers demonstrated that this type of grinding disc significantly mitigated defects caused by abrasive particles on the material surface, thereby enhancing the surface processing quality of the workpiece. Liu X et al. [11] fabricated solid abrasive grinding discs using SiO_2_ and carried out grinding and polishing experiments on single-crystal silicon wafers and microcrystalline glass, achieving surfaces with precision levels surpassing those obtained through free abrasive processing. Sun Yuli et al. from Nanjing University of Aeronautics and Astronautics [12] prepared frozen solid abrasive grinding discs utilizing SiO_2_ and Al_2_O_3_ based on principles of solid abrasive machining and polishing techniques. Through their experiments involving microcrystalline glass, they attained ultra-smooth surfaces with a remarkable precision measurement of 0.293 nm, exceeding that achieved via free abrasive methods.

Through a comparative study of two processing methods, it has been determined that the solid abrasive grinding process achieves superior processing quality and removal efficiency, thereby gradually establishing itself as the predominant method in grinding applications. Enhancements to the grinding disc utilizing solid abrasives can result in improved surface quality during processing. However, existing research primarily concentrates on experiments involving the grinding disc itself, with insufficient discussion and analysis regarding the associated processing techniques within grinding experiments. In terms of abrasive selection, diamond abrasive grains exhibit characteristics such as high hardness, exceptional strength, excellent wear resistance, elevated processing efficiency, and environmental friendliness. These attributes render them particularly suitable for machining hard and brittle materials. This paper will employ solid diamond abrasive grains as the cutting tool while integrating a planetary grinding motion mode to develop a mathematical model for the grinding process. Furthermore, analyses and selections of process parameters will be conducted. Two sets of experimental parameters—namely abrasive grain size and grinding pressure—will be designed to investigate both surface quality and subsurface damage in K9 glass following solid abrasive grinding.

## 2. Investigation of the K9 Glass Grinding Removal Model and Its Impact on Surface Quality (Mathematical Model for Planar Grinding Motion)

### 2.1. Development of a Mathematical Model for Grinding Trajectory

**Figure 2:**
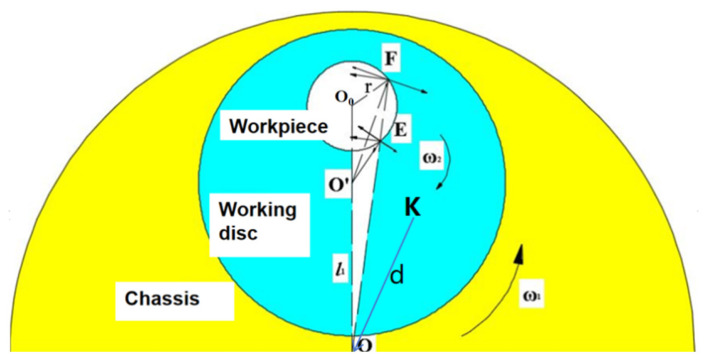
Grinding schematic diagram.

**Figure 3:**
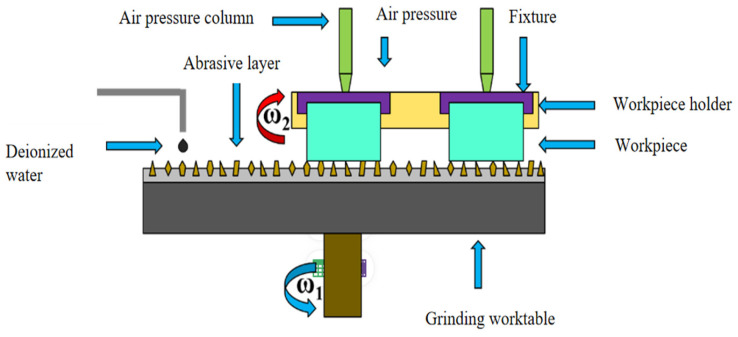
Geometric representation of the workpiece disc.

The free-abrasive processing device for optical components [13,14] includes a base, workpiece disc, grinding disc, and grinding fluid delivery system. The grinding equipment employed in this study is the OPG600 grinder, with its manufacturer being Qinchuan Machine Tool & Tool Group Corp. (Baoji, China) The grinding fluid, composed of abrasives and deionized water, allows abrasive particles to move and cut between the grinding disc and workpiece to remove material. During the grinding process of optical elements, both the workpiece disc and the worktable rotate around their respective centres in opposite directions. This motion can be effectively modelled as a planetary motion. Each individual workpiece rotates around the centre *O*_0_ of the workpiece disc with an angular velocity denoted by ω. The chassis rotates around point O at a speed of $w_{1}$, the working disc rotates around point O’ at a speed of $w_{2}$, the distance between O and O’ is l, and the radius of the workpiece is set as r. The movements of all four workpieces are identical and can be simplified to represent a single workpiece, as illustrated in Figure 2 and Figure 3.

### 2.2. Establishment of the Absolute Trajectory

According to Figure 2, a coordinate system is established with the centre of the grinding disc as the origin for a point K located on the surface of the workpiece. The absolute position equation for point K on the grinding disc can be derived as follows:

(1) $$\left\{\begin{array}{c} K_{0x}=d\cos\left(w_{1}t\right)+r_{d}\cos\left(-w_{1}t\pm w_{2}t+\theta_{0}\right)\\ K_{0y}=d\sin\left(w_{1}t\right)+r_{d}\sin\left(-w_{1}t\pm w_{2}t+\theta_{0}\right) \end{array}\right..$$

In the equation, *K*_0*x*_ represents the absolute x-coordinate value of point K; *K*_0*y*_ represents the absolute y-coordinate value of point K; θ_0_ indicates the initial angle of point K on the workpiece surface relative to the centre of the grinding disc; *t* refers to processing time; *r_d_* signifies the distance from point K on the workpiece to the centre of the workpiece disc; and *d* represents the eccentric distance between the centre of the workpiece disc and that of the grinding disc. $w_{1}$ represents the speed of the chassis rotating around point O, and $w_{2}$ represents the speed of the working disc rotating around point O’.

We set *d* to 55 mm, *r_d_* to 65 mm, the time *t* to 3 s, and the initial angle θ_0_ to 0°. Based on actual processing equipment specifications, the maximum rotational speed of the workpiece disc is set at 120 r/min, while that of the grinding disc is set at 600 r/min. The speed ratio *n*_2_:*n*_1_ is established at values of 0.2, 0.25, 0.33, 0.4, 0.6, 0.8, 1, and 2. With an initial setting for the workpiece disc speed at 100 r/min, trajectory simulation produces the absolute trajectory of point K on the workpiece as illustrated in the accompanying figure.

**Figure 4:**
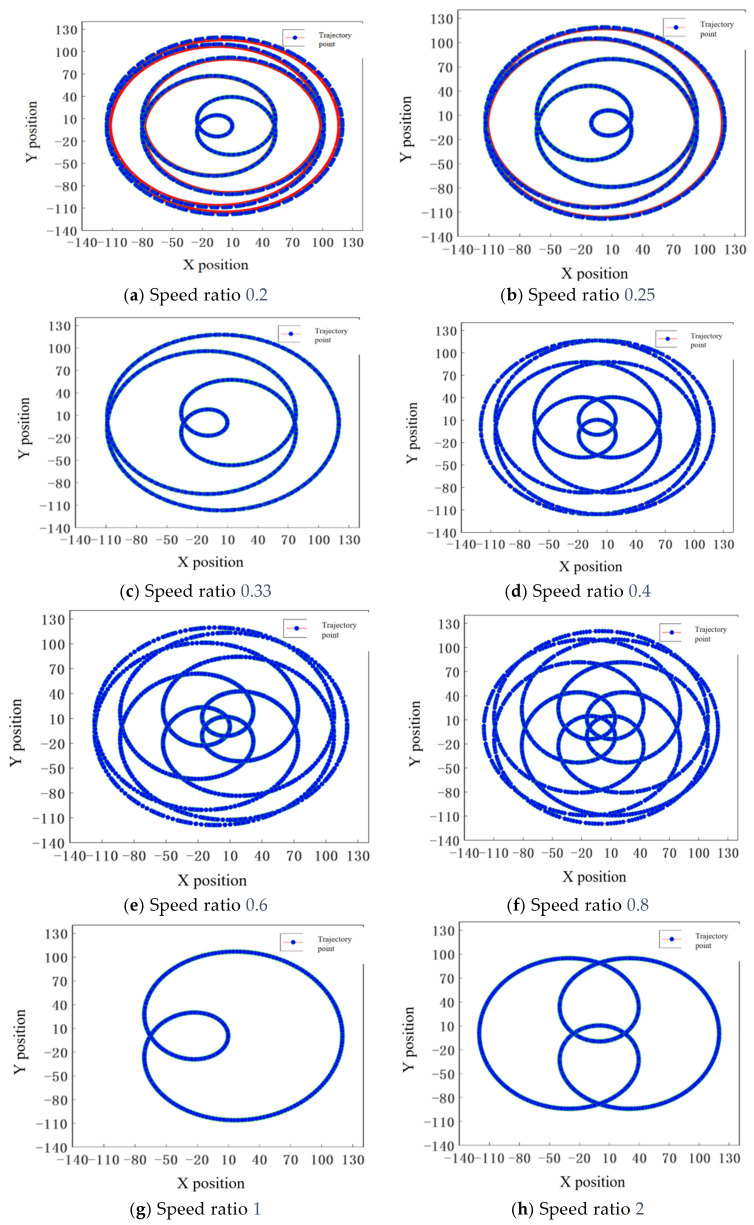
Trajectory diagram of point K at different speed ratios.

When *n*_2_:*n*_1_ > 1, the trajectory points diversify with increasing speed ratio. The workpiece disc speed is set between 60~100 r/min, and the speed ratio of the workpiece disc to the grinding disc is set between 0.6~1, limiting the grinding disc speed to 200 r/min. As shown in Figure 4c,d, this ensures sufficient coverage of the grinding disc area, reduces repeated grinding of specific regions, and minimizes wear of abrasive grains, especially smaller ones.

### 2.3. Establishment of the Relative Trajectory

Similarly, the position of point K relative to the chassis during the grinding process can be obtained as follows:

(2) $$\left\{\begin{array}{c} K_{x}={O^{\prime }}_{x}+r\cos\left(w_{2}t+\varphi_{0}\right)\\ K_{y}={O^{\prime }}_{y}-r\sin\left(w_{2}t+\varphi_{0}\right) \end{array}\right..$$

In the equation, *K_x_* represents the x-coordinate value of point K; *K_y_* denotes the y-coordinate value of point K; φ_0_ indicates the initial angle of point K on the workpiece surface relative to *OO′*; *t* signifies processing time; and *r* refers to the distance from point K on the workpiece to the centre of the workpiece disc.

In planar grinding, the layer of the workpiece is removed through the grinding action that occurs between the grinding disc and the surface of the workpiece [15]. This interaction induces relative motion between a point on the workpiece surface and the rotating grinding disc. By translating and transforming the coordinates of point K, we can derive the following equation:

(3) $$\left({K_{x}}^{\prime },{K_{y}}^{\prime }\right)=\left(K_{x}-O_{x},K_{y}-O_{y}\right)\left(\begin{array}{cc} \cos\theta & \sin\theta \\ -\sin\theta & \cos\theta \end{array}\right).$$

In the equation, θ represents the rotation angle of the grinding disc; *O_x_* denotes the x-coordinate value of point O, which is the centre of the grinding disc; and *O_y_* indicates the y-coordinate value of point O. Substituting Equation (2) into Equation (3) yields the following:

(4) $$\begin{array}{l} \left({K_{x}}^{\prime },{K_{y}}^{\prime }\right)=\\ \left(O\prime_{x}+r\cos\left(w_{2}t+\varphi_{0}\right)-O_{x},O\prime_{y}-r\sin\left(w_{2}t+\varphi_{0}\right)-O_{y}\right)\left(\begin{array}{cc} \cos w_{1}t & \sin w_{1}t\\ -\sin w_{1}t & \cos w_{1}t \end{array}\right) \end{array}.$$

Based on the formula, we set *d* to 55 mm, *r_d_* to 65 mm, *t* to 3 s, initial angle θ_0_ to 0°, and *ω*_2_:*ω*_1_ to 0.5 s, 1.5 s, 3 s, 5 s, 10 s, 20 s, 30 s, and 40 s. The grinding disc and workpiece disc rotate in opposite directions. With the workpiece disc speed initially set at 100 r/min, the relative trajectory of point K on the workpiece at different moments can be obtained through simulation.

Based on the established formula, we set the parameters as follows: *d* = 55 mm, *r_d_* = 65 mm, *t* = 3 s, initial angle θ_0_ = 0°, and *ω*_2_:*ω*_1_ at intervals of 0.5 s, 1.5 s, 3 s, 5 s, 10 s, 20 s, 30 s, and 40 s. The grinding disc and workpiece disc are configured to rotate in opposite directions. With the initial speed of the workpiece disc set at 100 r/min, a simulation can be conducted to obtain the relative trajectory of point K on the workpiece at various moments.

**Figure 5:**
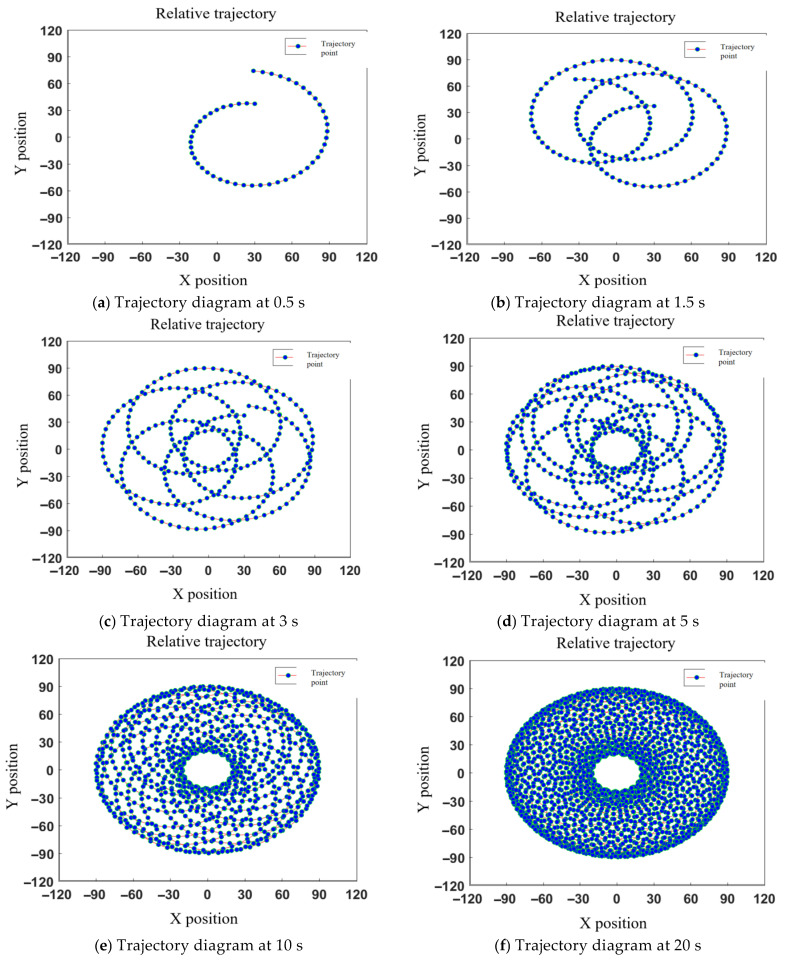
Relative trajectory diagram of point K at different moments.

The figure illustrates the simulated relative motion trajectory of a single point K, with a radius of 35 mm, at various moments in time (t) and an initial angle of θ = 0°. In Figure 5f, they appear particularly dense, indicating periodic motion under ideal conditions. Points located at different radii on the workpiece exhibit distinct trajectories due to variations in their respective r values. The motion period of point K corresponds to the least common multiple of the rotation periods of both the grinding disc and the workpiece disc. By superimposing trajectories from points with varying radii and phases, one can effectively simulate material removal paths, thereby facilitating a deeper understanding of abrasive grinding fundamentals.

### 2.4. Refinement of Surface Damage Detection

#### The Influence of Abrasive Grain Size on Surface Roughness

**Figure 6:**
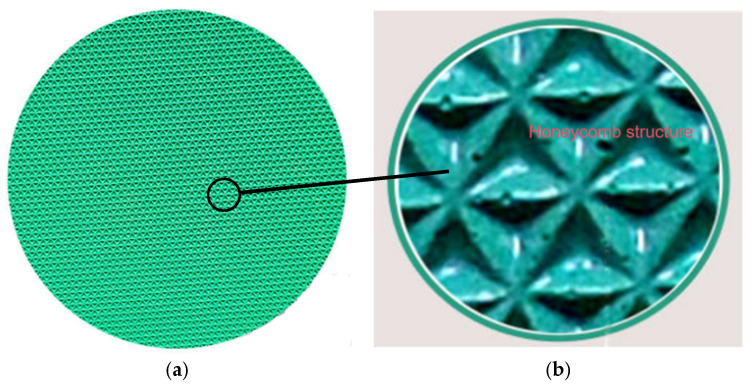
(**a**) Schematic diagram of the grinding disc. (**b**) Partial view of the grinding disc.

To investigate the grinding effects of various abrasive grain sizes on K9 optical glass, grinding discs with grain sizes of 28 μm, 20 μm, 14 μm, and 10 μm were used. The grinding disc illustrated in Figure 6 features a honeycomb structure with diamond grains embedded within its polymer matrix, ensuring continuous abrasive engagement throughout the grinding process [16].

After pre-treatment, the workpiece exhibits a diameter of Φ208.9 mm and weights of 7.0666 g, 7.0406 g, 7.0662 g, and 7.0446 g, demonstrating a weight variation of less than 0.37%. This indicates that the weights can be considered equivalent. The experimental parameters are shown in Table 1.

After a grinding duration of 10 min, the surface was analyzed using a laser confocal microscope, and the resulting topography is presented in the figure [17]. The laser confocal microscope employed in the experiment (for surface morphology observation) is manufactured by Nanjing Kaisimai Technology Co., Ltd. (Nanjing, China).

**Figure 7:**
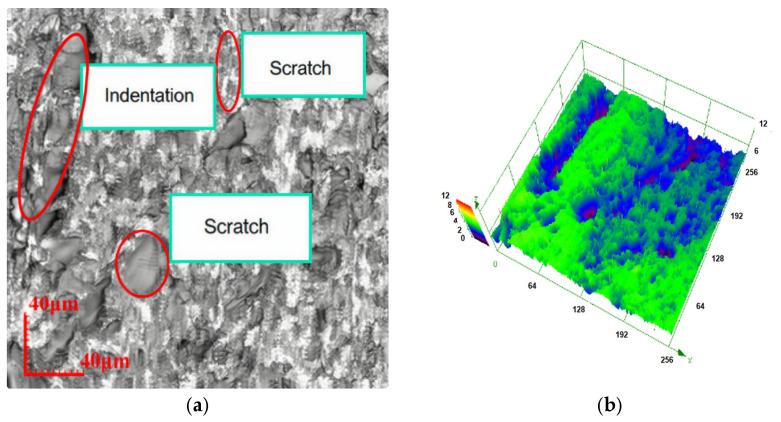

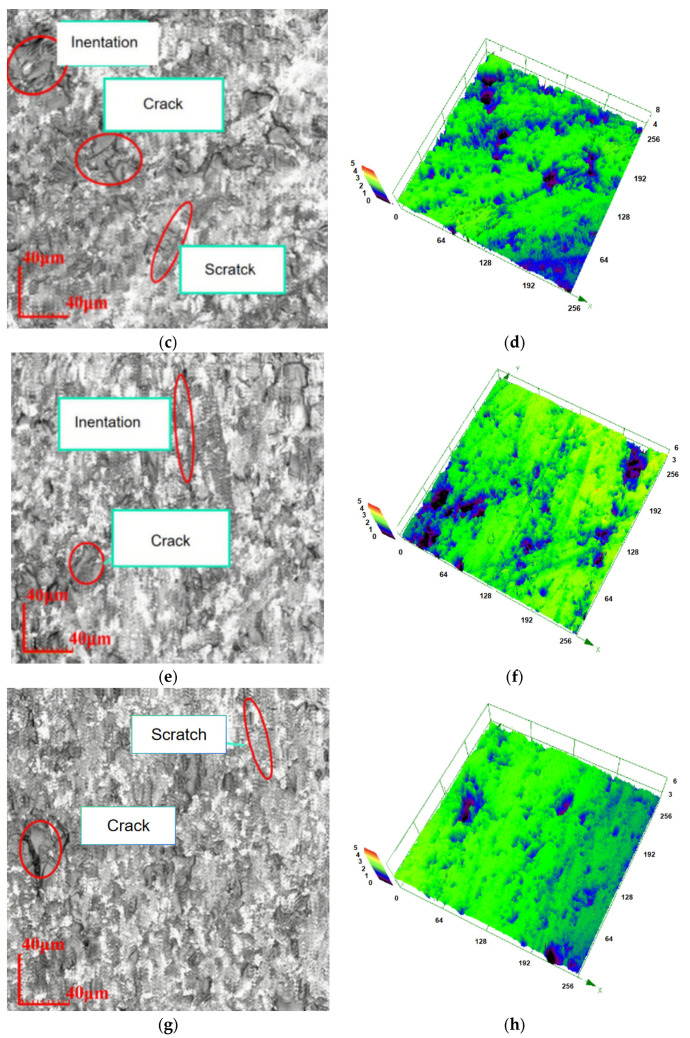
Surface topographies for different abrasive grain sizes. (**a**) Two-dimensional surface after grinding with 28 μm grinding disc. (**b**) Three-dimensional surface after grinding with 28 μm grinding disc. (**c**) Two-dimensional surface after grinding with 20 μm grinding disc. (**d**) Three-dimensional surface after grinding with 20 μm grinding disc. (**e**) Two-dimensional surface after grinding with 14 μm grinding disc. (**f**) Three-dimensional surface after grinding with 14 μm grinding disc. (**g**) Two-dimensional surface after grinding with 10 μm grinding disc. (**h**) Three-dimensional surface after grinding with 10 μm grinding disc.

With consistent grinding disc speed, grinding pressure, and workpiece velocity, a reduction in abrasive grain size leads to fewer surface indentations and enhances the overall quality of the grinding process. Figure 7a,c,e show the 2D images of the sample surface morphology, Figure 7b,d,f show the 3D images at the corresponding positions. As illustrated in Figure 7g, the occurrence of broken pits and indentations on the glass surface is markedly lower compared to those produced by Figure 7a,c,e.

### 2.5. The Influence of Abrasive Grain Size on Material Removal

**Figure 8:**
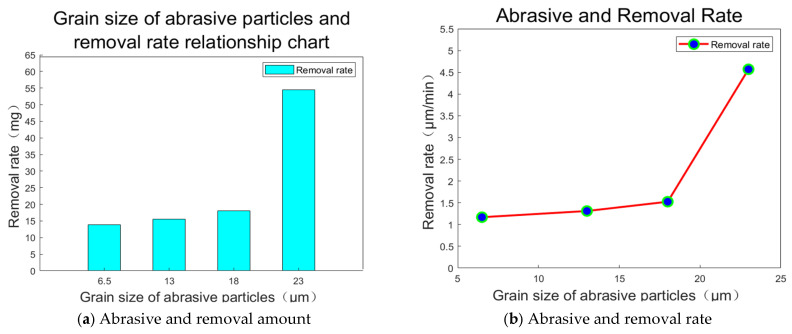
Relationship between abrasive grain size and workpiece removal.

The weight of the ground samples was determined using an electronic balance, and the data presented in Table 2 were obtained through calculations:

A curve chart can be drawn as shown in Figure 8:

The effect of material removal is positively correlated with the grain size of the grinding disc. Larger diamond abrasive grains possess greater facet surface areas, which increases the contact area with the sample surface and enhances the material removal action. Additionally, larger grains feature sharper cutting edges that facilitate deeper penetration into the workpiece. Consequently, both the amount and rate of material removal increase as diamond grain sizes become larger.

#### 2.5.1. The Influence of Grinding Pressure on Surface Roughness

**Figure 9:**
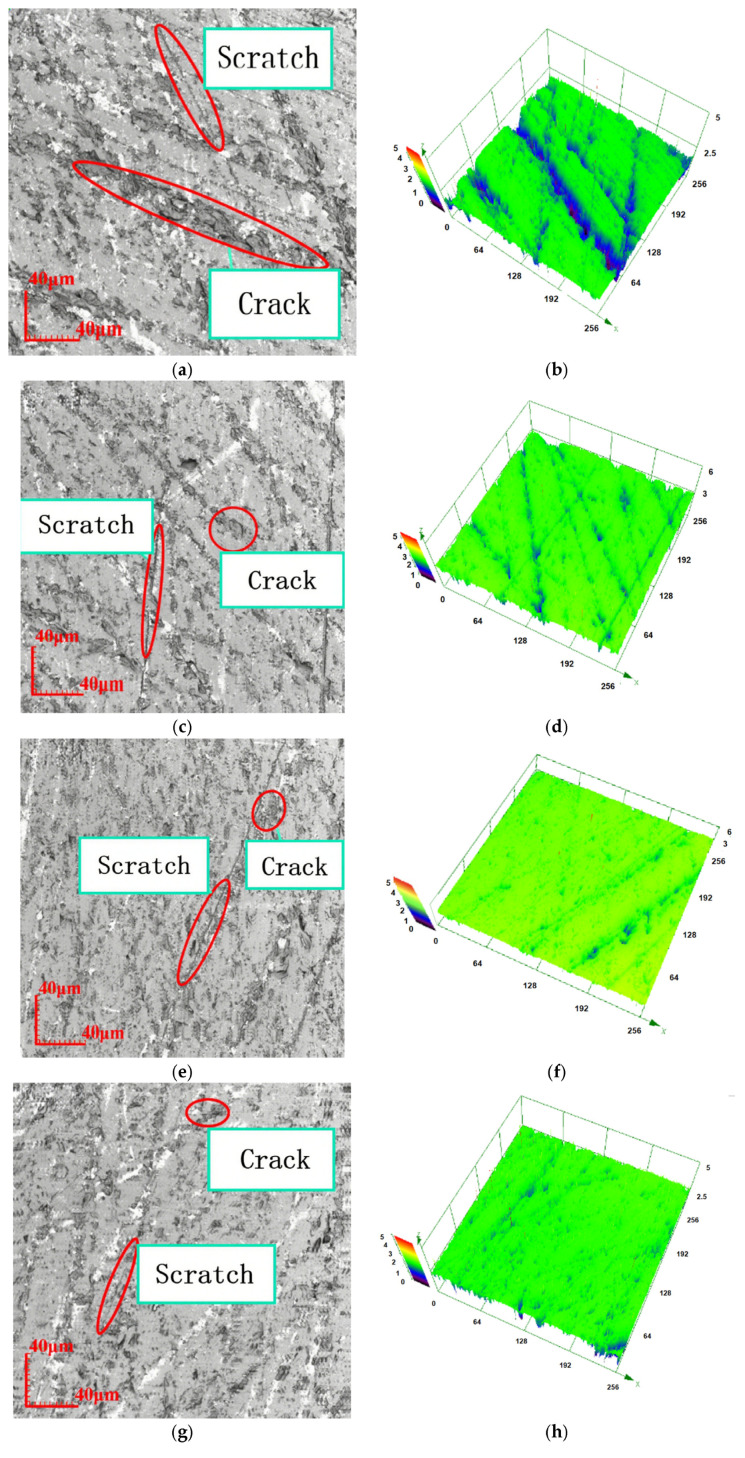
Surface topography at different grinding pressures. (**a**) Two-dimensional surface at 13.61 × 10^−3^ MPa. (**b**) Three-dimensional surface at 13.61 × 10^−3^ MPa. (**c**) Two-dimensional surface at 10.42 × 10^−3^ MPa. (**d**) Three-dimensional surface at 10.42 × 10^−3^ MPa. (**e**) Two-dimensional surface at 7.24 × 10^−3^ MPa. (**f**) Three-dimensional surface at 7.24 × 10^−3^ MPa. (**g**) Two-dimensional surface at 4.05 × 10^−3^ MPa. (**h**) Three-dimensional surface at 4.05 × 10^−3^ MPa.

To investigate the grinding effects of varying pressures on K9 optical glass, grinding pressures of 1 N, 2 N, 3 N, and 4 N were employed. The workpiece, following pre-treatment, has a diameter of Φ209 mm. The pressures exerted on the four samples were calculated as follows (Table 3):

We selected a 20 μm grinding disc; the measurement results of the grinding disc are presented in Figure 9. The blue region in the right-hand figure indicates the area that has sustained damage following the grinding process, while the green region represents the surface of the workpiece that remains intact.

From Figure 9, it is clear that maintaining a constant grinding disc speed, abrasive grain size, and workpiece speed while gradually reducing the grinding pressure results in finer grooves and scratches on the glass surface. As a consequence, there is a significant improvement in surface quality, which leads to enhanced grinding performance.

**Figure 10:**
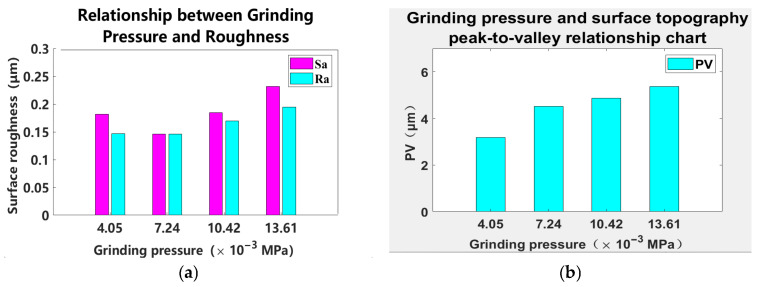
Relationship between grinding pressure and surface quality. (**a**) Relationship between grinding pressure and roughness. (**b**) Relationship between grinding pressure and surface PV.

Based on the data in Table 4, Figure 10 is constructed:

As illustrated in Figure 10a, the surface roughness Ra of the workpiece exhibits an increasing trend with elevated grinding pressure. This phenomenon can be attributed to the sharp edges of the abrasive grains, which create marks of varying depths on the sample surface when subjected to pressure [18]. An increase in pressure enhances the number of effective cutting grains, resulting in greater surface roughness. Furthermore, higher pressure facilitates complete contact between pure water and the glass surface, leading to the formation of a softened layer that aids in material removal by abrasive grains. The cutting edges of these grains penetrate more deeply into the glass substrate, thereby creating larger valleys and contributing to an increased surface PV.

##### The Influence of Grinding Pressure on Material Removal Rate

Based on Preston’s equation [19], the material removal rate over a time period T can be derived from the velocity and pressure at a specific point on the workpiece, as illustrated in Equation (5):

(5) $$\Delta z\left(x,y\right)=K\int_{0}^{T}V\left(x,y,t\right)P\left(x,y,t\right)dt.$$

**Figure 11:**
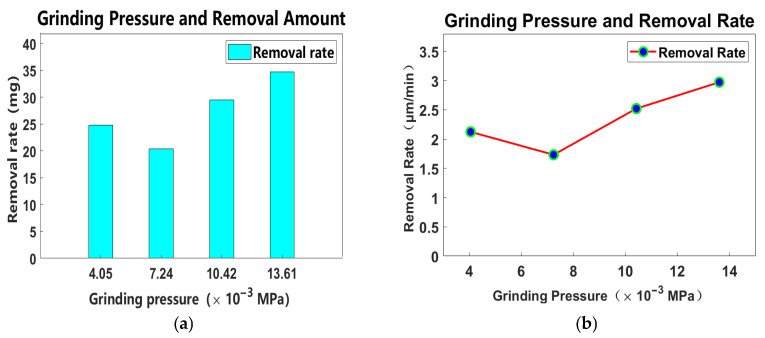
Relationship between grinding pressure and workpiece removal. (**a**) Grinding pressure and removal amount. (**b**) Grinding pressure and removal rate.

The measured data after grinding is shown in Table 5:

A curve chart can be illustrated as depicted in Figure 11:

As illustrated in Figure 11, from the chart of “Grinding Pressure and Removal Rate”, the following conclusion can be drawn regarding the relationship between grinding pressure and workpiece removal rate: overall, the workpiece removal rate shows an upward trend with the increase in grinding pressure. Specifically, within the grinding pressure range of 4.05 × 10^−3^ MPa to 7.24 × 10^−3^ MPa, the removal rate fluctuates slightly (with a minor decrease). However, when the grinding pressure exceeds 7.24 × 10^−3^ MPa, the removal rate increases significantly and continuously as the pressure rises. This indicates that under relatively high grinding pressure conditions, increasing the pressure can effectively promote the removal of workpiece material and improve processing efficiency.

#### 2.5.2. Comparison of Abrasive Grain Size and Grinding Pressure

**Figure 12:**
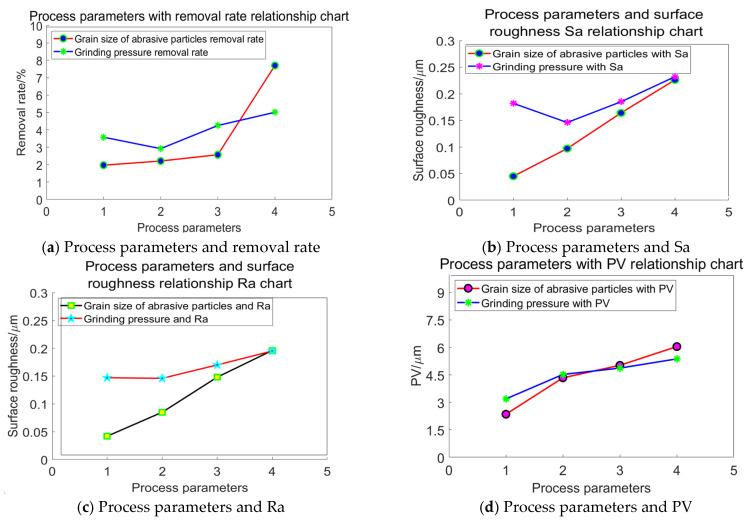
Comparison of abrasive grain size and grinding pressure effects.

In the grinding process, the base speed is 150 r·min^−1^ and the workpiece disc speed is 100 r·min^−1^. When examining the relationship between grinding pressure and abrasive particle size, process parameters 1 to 4 in Figure 12 correspond to the aforementioned four combinations of varying pressures and different abrasive particle sizes. The process parameters are arranged in ascending order according to the abrasive particle size and grinding parameters mentioned above, respectively. Specific parameters are shown in Table 6.

As illustrated in Figure 12a, the trend of material removal rate in relation to abrasive grain size is more pronounced than that associated with grinding pressure. The curve representing grinding pressure typically exhibits an upward trajectory as the pressure increases. In contrast, the material removal rate rises with larger grain sizes, demonstrating a sharp increase within a specific range. This indicates that larger grain sizes exert a more significant influence on material removal compared to grinding pressure.

In Figure 12b–d, it is evident that variations in surface quality attributable to grain size are considerably more substantial than those resulting from changes in grinding pressure, which displays a relatively mild trend. This observation suggests that grain size has a more marked effect on processing quality than fluctuations in grinding pressure. Consequently, when selecting parameters for processing, it is essential to consider the impacts of both grain size and pressure on surface quality.

Large-sized abrasive particles can enhance processing efficiency; however, they may lead to a deterioration in surface quality, characterized by high roughness and significant subsurface damage. In contrast, small-sized abrasive particles can markedly improve surface quality but result in lower processing efficiency and extended timeframes to achieve the desired accuracy. Consequently, it is essential to strike a balance during actual processing based on specific requirements: if efficiency is prioritized (as in the rough grinding stage), large-sized abrasive particles should be selected; conversely, if a high-precision surface is necessary (such as in the fine grinding or pre-polishing stages), small-sized abrasive particles are recommended. Additionally, any subsurface damage can be further mitigated through subsequent polishing processes.

### 2.6. Refinement of Grinding Process

This chapter primarily explores the grinding mechanism, the mathematical models associated with the grinding process, the optimization of grinding parameters, and the influence of abrasive grain size and grinding pressure on processing outcomes.

Mathematical modelling was conducted for planetary grinding utilizing bonded abrasives. Simulations were performed to analyze both the absolute and relative trajectories of the workpiece in relation to the grinding disc, as well as to investigate the effects of varying speed ratios on surface uniformity. The experimental range for speed ratios was established between 0.6 and 1. Grinding and polishing experiments conducted on K9 glass resulted in surface qualities characterized by Rq and Ra values within 0.35 nm, with a planar height difference not exceeding 10 nm. The parameters obtained from simulations were validated through experimental methods, thereby providing standard samples for subsequent grinding experiments.

Grinding experiments were conducted with abrasive grain size and grinding pressure as the primary variables. The results indicated that removal efficiency is directly proportional to grain size, whereas surface quality (Ra, Sa, PV) exhibits an inverse relationship with grain size. Additionally, both removal efficiency and surface quality are positively correlated with grinding pressure. A comparative analysis of these two parameters revealed that grain size exerts a more significant influence on processing efficiency and surface quality.

## 3. Model for the Relationship Between Subsurface Damage Depth and Other Variables

### 3.1. Subsurface Damage Model

**Figure 13:**
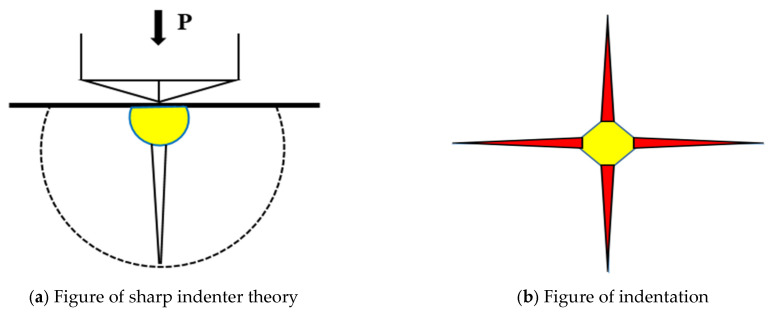
Sharp indenter model.

The process of removing glass material using diamond grains is conceptualized as either indentation or scratching by a sharp or blunt indenter, as illustrated in Figure 13a. Upon indentation, the glass material develops radial cracks, as depicted in Figure 13b.

**Figure 14:**
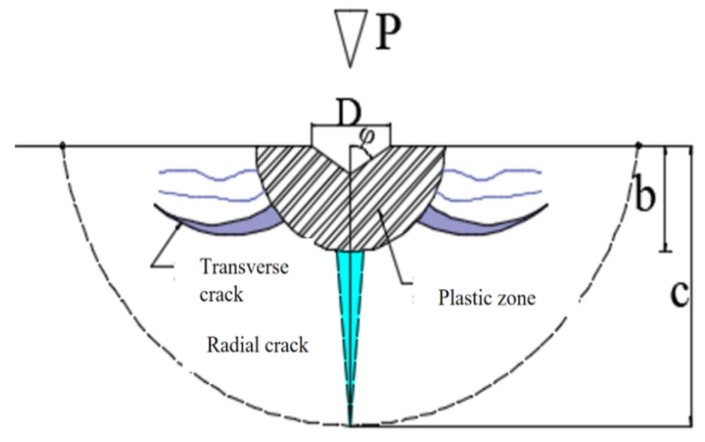
Indenter penetration area.

**Figure 15:**
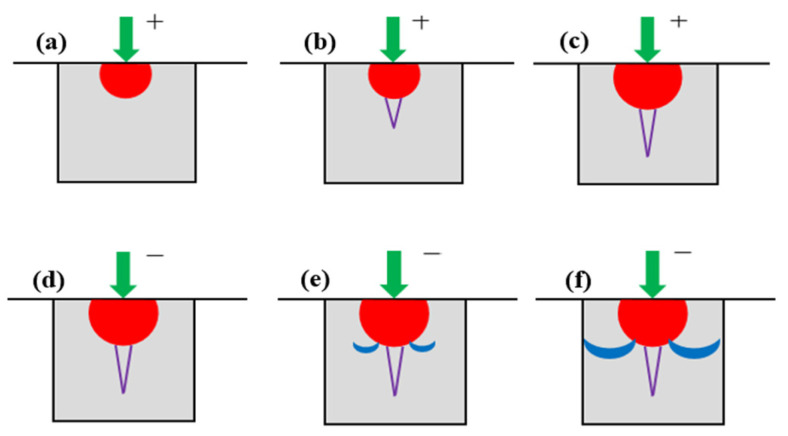
Schematic of Crack Development Under the Action of a Sharp Indenter. (**a**) When the sharp indenter presses into the glass surface, plastic and irreversible deformation occurs in the contact area, accompanied by microcracks. (**b**) Damage propagates within the irreversible deformation zone, resulting in the generation of radial cracks. (**c**) Radial cracks extend further downward. (**d**) When the indenter begins to retract, the propagation of radial cracks below this deformation zone ceases and begins to close. (**e**) After the indenter is fully withdrawn, residual stresses cause radial cracks to extend and generate transverse cracks in the lower part of the deformation zone. (**f**) After the indenter completely separates from the glass surface, the propagation of cracks ceases.

According to Lawn B [20], as the applied load P increases, both radial and lateral cracks develop beneath the indenter in the glass due to the interplay of elastic and plastic stress fields. As illustrated in Figure 14, radial cracks propagate downward; with greater penetration depth, these cracks exhibit more intense propagation, which signifies the subsurface damage depth (SSD). Lateral cracks emerge during material removal and extend along the surface, thereby contributing to an increase in surface roughness characterized by its peak-to-valley (PV) value. Figure 15 illustrates the development process of these crack systems [21]. 

Figure 15a The tip of the indenter is pressed into the material, resulting in plastic and irreversible deformation within the contact area between the two.

Figure 15b Once the load applied by the indenter reaches a critical threshold, damage induced by the indenter begins to propagate and grow within the zone of irreversible deformation. At this juncture, radial cracks are generated on the tensile mid-plane beneath this zone and at locations where stress concentration occurs.

Figure 15c As load P continues to increase, these radial cracks further develop downward.

Figure 15d When the indenter starts to retract, load P is gradually released. During this process, the elastic region in the contact area between the indented portion of the indenter and material recovers its deformation; concurrently, growth of radial cracks below this deformation zone ceases and they begin to close. However, residual stresses generated on the glass surface during indentation remain in an open state.

Figure 15e Upon complete withdrawal of the indenter from the glass surface, residual stresses on that surface become predominant. This leads to further extension and propagation of radial cracks located within areas affected by irreversible deformation. Simultaneously, disc-shaped transverse cracks extending outward form near lower regions of this deformation zone.

Figure 15f Once separation between the indenter and glass surface is fully achieved, crack propagation halts. At this stage, an area enclosed by both transverse and radial cracks can be conceptualized as a semi-cake-shaped region centered around the point of indentation made by the indenter.

We established a mathematical relationship between transverse and radial cracks to elucidate the variation in surface roughness (SR) and subsurface damage depth (SSD). Given that subsurface damage is challenging to measure, the proposed model, along with SR, can effectively predict the propagation depth of subsurface cracks (SSD).

Based on the theoretical model of subsurface damage, the correlation between surface roughness and the depth of subsurface damage is established as follows:

(6) $$\alpha_{k}=0.027+0.09\left(m-\frac{1}{3}\right).$$

The theoretical formula for calculating the depth of radial cracks [22] is as follows:

(7) $$c_{m}={\alpha_{k}}^{\frac{2}{3}}{\left(\frac{E}{H_{V}}\right)}^{\frac{2\left(1-m\right)}{3}}{\left(\cot\varphi \right)}^{\frac{4}{9}}{\left(\frac{F_{n}}{K_{IC}}\right)}^{\frac{2}{3}}.$$

In the formula, *c_m_* represents the radial crack depth; *F_n_* denotes the normal load, which is the force applied by the indenter; *E* refers to the elastic modulus; φ indicates the tip angle of the Vickers indenter; *H_V_* signifies Vickers hardness; *K_IC_* is a parameter for fracture toughness; *m* is a dimensionless constant that ranges between 1/2 and 1/3; and α*_k_* is a dimensionless parameter related to m.

The calculation formula for transverse crack depth is as follows:

(8) $$h=0.43{\left(\sin\varphi \right)}^{\frac{1}{2}}{\left(\cos\varphi \right)}^{\frac{1}{3}}{\left(\frac{E}{H_{V}}\right)}^{m}{\left(\frac{F_{n}}{H_{V}}\right)}^{\frac{1}{2}}.$$

In the formula, *h* represents the depth of the transverse crack.

### 3.2. HF Chemical Etching Method

The subsurface damage depth of K9 glass components, following grinding with various abrasive particle sizes and grinding pressures, was assessed using the HF chemical etching method, which is based on the principle of differential corrosion rates. In this experiment, to ensure the accuracy of the results obtained, both the workpiece and reference piece were subjected to identical experimental conditions. The detailed calculation principles are outlined as follows:

(9) $$\left\{\begin{array}{c} \Delta m=m_{0}-m_{n}\\ \Delta m_{BM}={m_{0}}_{BM}-{m_{n}}_{BM}\\ \Delta m^{\prime }=\Delta m-\frac{\Delta m_{BM}}{2} \end{array}\right..$$

In the formula, *m*_0_ represents the mass of the processed sample before corrosion, and the masses after corrosion for one time period are *m*_1_, *m*_2_, and *m_n_*; *m_n_* indicates the mass of the processed sample after n time periods of corrosion; Δ*m* represents the double-sided corrosion amount of the processed piece; ${m_{0}}_{BM}$ represents the mass of the processed sample before corrosion, and the masses after corrosion for one time period are ${m_{1}}_{BM}$, ${m_{2}}_{BM}$, ${m_{n}}_{BM};\;{m_{n}}_{BM}$ indicates the mass of the processed sample after n time periods of corrosion; $\Delta m_{BM}$ represents the double-sided corrosion amount of the reference piece; Δ*h_n_* represents the double-sided corrosion depth after n time periods; Δ*m′* represents the corrosion amount of the processing surface of the processed piece; and *H* represents the thickness of the glass before corrosion.

For the single-sided corrosion depth Δ*h_n_* within the nth time period, the chemical equation is as follows:

(10) $$\frac{m_{0}}{H}=\frac{\Delta m^{\prime }}{\Delta h_{n}}.$$

From the above equation, the corrosion rate v within the n th time period can be obtained:

(11) $$v=\frac{\Delta h_{n}}{t}.$$

In the formula, *t* represents the time interval of corrosion; $v_{smp}$ represent the sample speed; and $v_{BM}$ represents the corrosion rate of the reference specimen. The differential corrosion rate $v_{Diff}$ can be obtained as follows:

(12) $$v_{Diff}=v_{smp}-v_{BM}.$$

The differential corrosion acceleration can be obtained as follows:

(13) $$a_{Diff}=\frac{{v_{Diff}}_{n}-{v_{Diff}}_{n-1}}{t}.$$

The average corrosion rate, differential corrosion rate, and differential corrosion acceleration of the processed sample were determined using the aforementioned formula. When the differential acceleration of the sample aligns with the corrosion acceleration of the reference specimen, this moment is designated as the point at which the corrosive liquid reaches the substrate. Subsequently, the corrosion depth is calculated based on the average rate of the processed sample and is considered to represent the depth of the subsurface damage layer.

The specific experimental procedures are as follows:

(1) Pre-treatment of the sample:

The K9 glass sample was subjected to rough grinding, fine grinding, rough polishing, fine polishing, and then completely immersed in a 40% HF acid solution for 2 h to ensure no subsurface damage exists. The hydrofluoric acid (HF) employed in this experiment was manufactured by Laiyang Kangte New Material Co., Ltd. (Yantai, China)

The four process parameters are in Table 7.

(2) Weigh the sample:

After the sample is treated, it is ultrasonically cleaned with acetone, alcohol, and deionized water to remove surface impurities, and then dried with dry air. The weight is measured with a precision electronic balance (accuracy of 0.1 mg), and the original weight is recorded.

(3) Prepare BOE solutions with different volume ratios:

HF acid solution (40 wt%) and NH4F solution (40 wt%) are mixed in volume ratios of 1:20 to prepare BOE solutions.

(4) Corrode the sample in the BOE solution:

The processed K9 sample is completely immersed in the etching solution. The etching time is an important factor affecting the glass etching. If the etching time is too long, the glass removal will be substantial, which will affect the observation of subsurface damage morphology. If the etching time is too short, the experimental efficiency will be low.

(5) Weigh the sample after etching:

The etched sample is cleaned, and its weight is measured with a precision electronic balance.

### 3.3. Analysis of Subsurface Damage

#### 3.3.1. Detection of Subsurface Damage Depth Induced by Abrasive Grain Size

**Figure 16:**
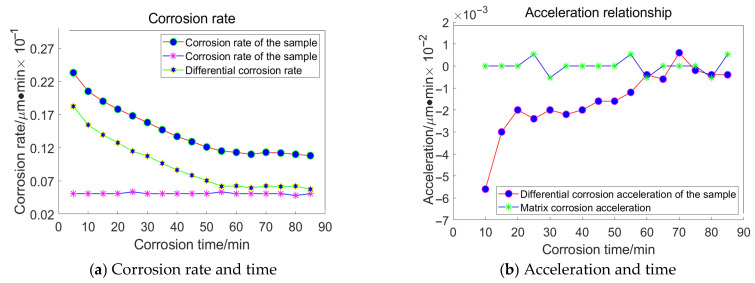
Relationship between Corrosion Duration and the Quantity of Workpiece Material Removed Utilizing 23 μm Abrasive Grains.

**Figure 17:**
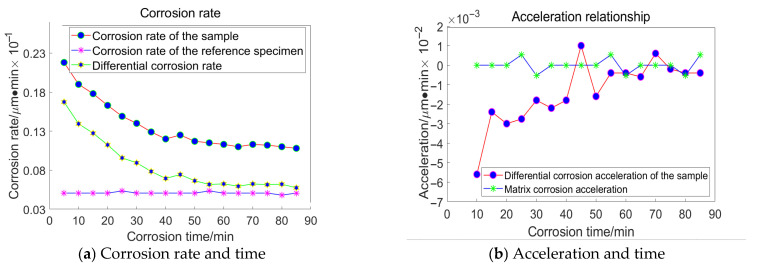
Relationship between Corrosion Duration and the Quantity of Workpiece Material Removed Utilizing 18 μm Abrasive Grains.

**Figure 18:**
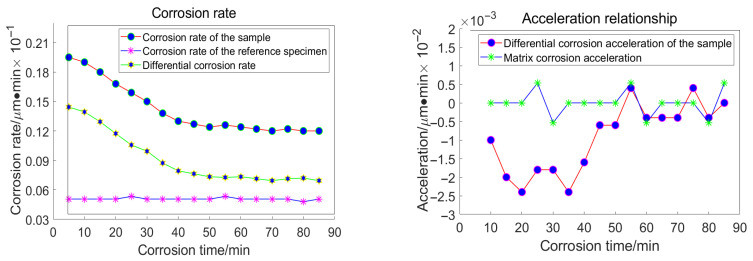
Relationship between Corrosion Duration and the Quantity of Workpiece Material Removed Utilizing 13 μm Abrasive Grains.

**Figure 19:**
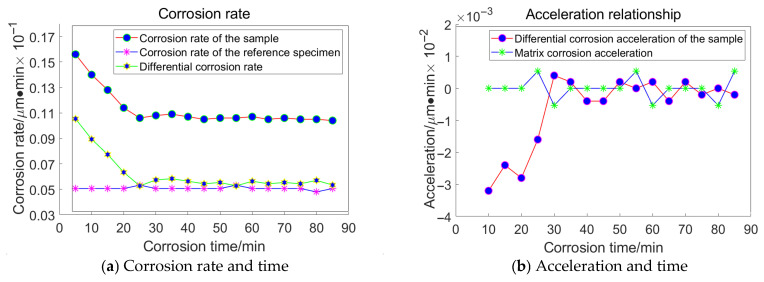
Relationship between Corrosion Duration and the Quantity of Workpiece Material Removed Utilizing 6.5 μm Abrasive Grains.

Based on the principles and procedures of HF chemical etching detection, For the grain size of 23 μm, the following data were acquired, as illustrated in Figure 16:

For the grain size of 18 μm, the following data were acquired, as illustrated in Figure 17:

For the grain size of 13 μm, the following data were acquired, as illustrated in Figure 18:

For the grain size of 6.5 μm, the following data were acquired, as illustrated in Figure 19:

Taking the specimen ground with a 23 μm grinding disc as an example, the experiment conducted on the substrate revealed that the average corrosion acceleration of the substrate approaches zero. At the 55th minute, the differential corrosion acceleration between the processed specimen and the substrate becomes comparable, indicating that corrosion has reached the substrate. In conjunction with Figure 16a, it can be concluded that the subsurface damage depth of K9 glass ground with a 23 μm grinding disc is approximately 4.5 μm. The corrosion inflexion points for the remaining three specimens were determined to be 65 min, 55 min, and 30 min, respectively, using the same methodology.

#### 3.3.2. Detection of Subsurface Damage Depth Induced by Grinding Pressure

**Figure 20:**
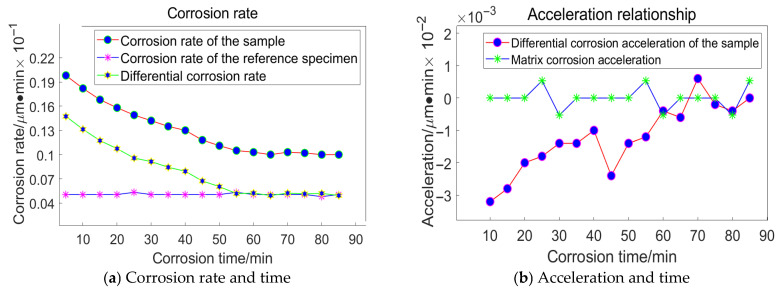
Corrosion time and removal relationship.

**Figure 21:**
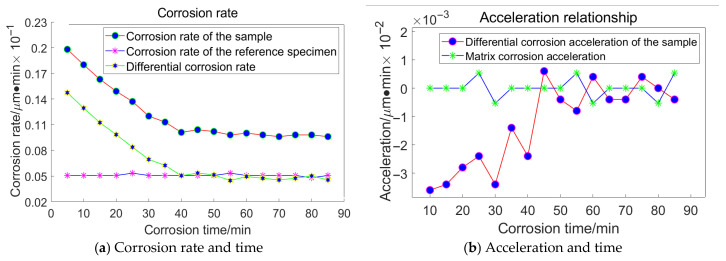
Corrosion time and removal relationship.

**Figure 22:**
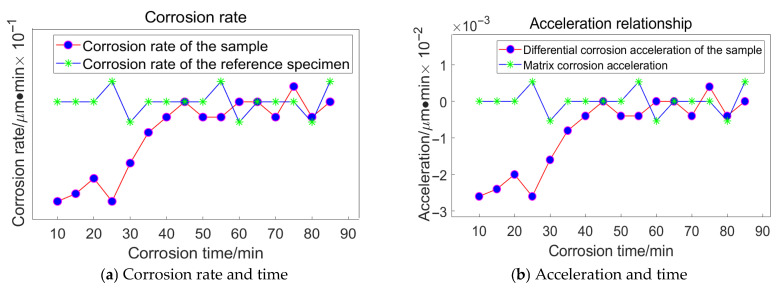
Corrosion time and removal relationship.

**Figure 23:**
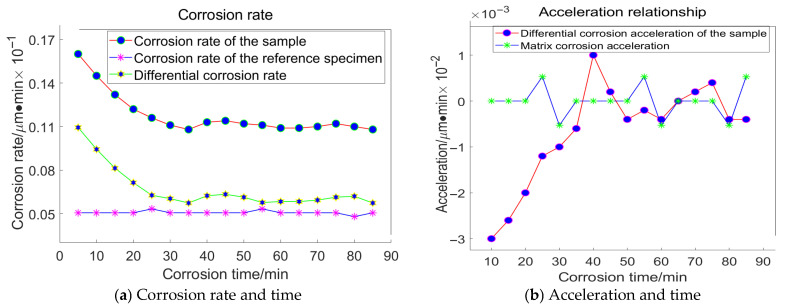
Corrosion time and removal relationship.

The processed data corresponding to a grinding pressure of 13.62 × 10^−3^ MPa is presented in Figure 20:

The processed data corresponding to a grinding pressure of 10.62 × 10^−3^ MPa is presented in Figure 21:

The processed data corresponding to a grinding pressure of 7.24 × 10^−3^ MPa is presented in Figure 22:

The processed data corresponding to a grinding pressure of 4.05 × 10^−3^ MPa is presented in Figure 23:

The corrosion inflexion points for the four specimens were determined to be 60 min, 53 min, 43 min, and 37 min, respectively, using a consistent methodology.

### 3.4. The Impact of Process Parameters on the Depth of Subsurface Damage

#### 3.4.1. The Correlation Between Abrasive Grain Size and Subsurface Damage Depth

**Figure 24:**
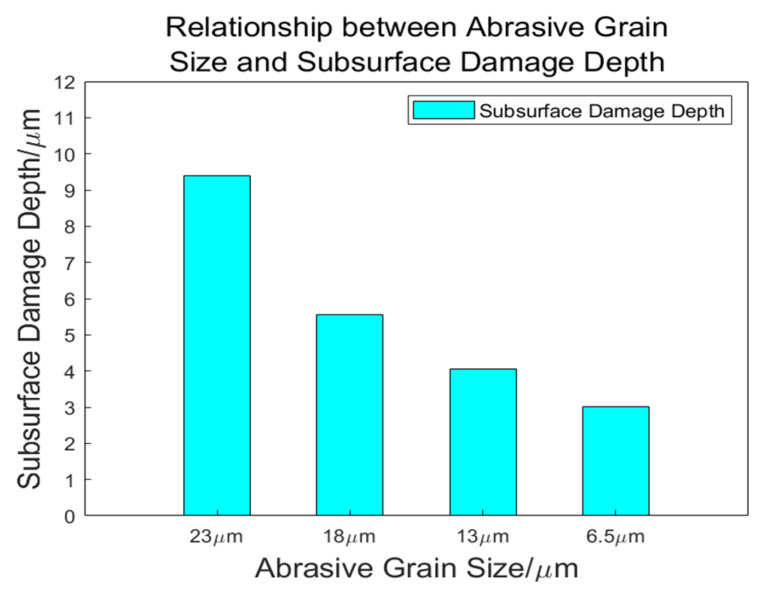
Relationship between abrasive grain size and subsurface damage depth.

Based on the analysis presented in the preceding section, the depth of subsurface damage is determined as follows (Table 8): 

A curve can be illustrated as depicted in Figure 24:

As the size of the abrasive grain decreases, the depth of subsurface damage also diminishes. During the grinding process, it is primarily the protruding portions of the abrasive grains that contribute to material removal from the specimen. A smaller grain size results in reduced scratching and ploughing effects on the specimen, leading to shallower penetration by individual grains and a decrease in subsurface cracking. Conversely, when larger grain sizes are employed, each abrasive grain bears a greater load, which intensifies surface scratching. As grinding progresses, although larger grains experience more wear over time, they still retain more material compared to their smaller counterparts; this ultimately leads to increased surface and subsurface damage.

In summary, during grinding operations, utilizing smaller grain sizes correlates with diminished levels of subsurface damage.

Based on Equation (7), the expression for the radial crack of an individual abrasive grain is derived as follows:

(14) $$c_{m}={\alpha_{k}}^{\frac{2}{3}}{\left(\frac{E}{H_{V}}\right)}^{\frac{2\left(1-m\right)}{3}}{\left(\cot\varphi \right)}^{\frac{4}{9}}{\left(\frac{F_{n}}{K_{IC}}\right)}^{\frac{2}{3}}.$$

For the size of abrasive grains, the normal force acting on the grinding particles can be determined based on the definition of hardness [23], as illustrated in the following equation:

(15) $$P_{i}=4H_{V}\tan^{2}\varphi_{i}d_{i}.$$

In the formula, *P_i_* represents the normal force exerted on the abrasive grain; *H_V_* denotes the hardness of the abrasive grain size; φ indicates the angle of the abrasive grain; and *d_i_* refers to the depth at which the abrasive grain is pressed into the specimen.

**Figure 25:**
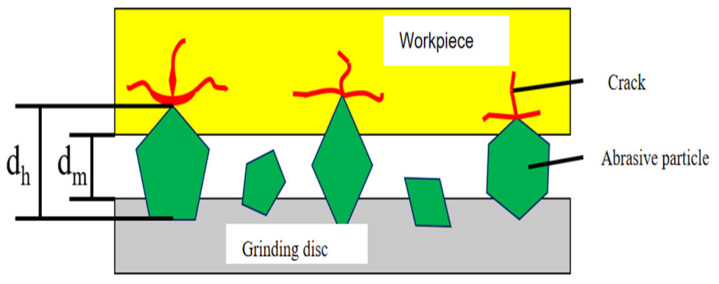
The positional relationship between the abrasive particle, the grinding disc, and the workpiece.

From Equation (14), it is evident that the depths of both radial and transverse cracks are directly proportional to the extent of abrasive grain penetration into the workpiece. This penetration depth is influenced by the size of the abrasive grains, as illustrated in Figure 25:

Based on the figure presented above, the correlation between abrasive grain size and penetration depth can be delineated as follows:

(16) $$d_{i}=d_{h}-d_{m}.$$

In the formula, *d_h_* represents the diameter of the abrasive grain; *d_m_* denotes the distance between the grinding disc and the workpiece; and *d_i_* indicates the depth to which the abrasive grain is pressed into the specimen.

By substituting Equations (15) and (16) into Equation (14), the relationship between a single abrasive grain and the depth of radial cracks can be derived, as expressed in the following equation:

(17) $$c_{m}={\alpha_{k}}^{\frac{2}{3}}{\left(\frac{E}{H_{V}}\right)}^{\frac{2\left(1-m\right)}{3}}{\left(\cot\varphi \right)}^{\frac{4}{9}}{\left(\frac{4H_{V}\tan^{2}\varphi_{i}\left(d_{h}-d_{m}\right)}{K_{IC}}\right)}^{\frac{2}{3}}.$$

Considering the continuous wear of abrasive grains during grinding, the value keeps changing. Combining Equation (15), the relationship between radial crack depth and grinding pressure during grinding is obtained.

In the formula, *F_ni_* represents the normal force exerted on the i-th abrasive grain, while *s* denotes the number of effective abrasive grains present in the grinding disc.

The relationship between an individual abrasive grain and the depth of radial cracks can be derived, as illustrated in the following equation:

(18) $${c_{m}}_{r}={\alpha_{k}}^{\frac{2}{3}}{\left(\frac{E}{H_{V}}\right)}^{\frac{2\left(1-m\right)}{3}}\int_{\varphi_{1}}^{\varphi_{2}}{\left(\cot\varphi_{i}\right)}^{\frac{4}{9}}{\left(\frac{4H_{V}\tan^{2}\varphi_{i}\sum_{1}^{s}\left(d_{hi}-{d_{m}}_{i}\right)}{K_{IC}}\right)}^{\frac{2}{3}}.$$

In the formula, *c_mr_* represents the radial crack depth generated during the grinding process; φ_1_ denotes the initial sharpness angle of the abrasive grain; φ_2_ indicates the final sharpness angle of the abrasive grain; and *s* refers to the number of effective abrasive grains present in the grinding disc.

Refining the expression, we arrive at the following:

(19) $${c_{m}}_{r}={\alpha_{k}}^{\frac{2}{3}}{\left(\frac{E}{H_{V}}\right)}^{\frac{2\left(1-m\right)}{3}}\int_{\varphi_{1}}^{\varphi_{2}}{\left(\frac{4H_{V}\sum_{1}^{s}\left(d_{hi}-{d_{m}}_{i}\right)}{K_{IC}}\right)}^{\frac{2}{3}}.$$

**Figure 26:**
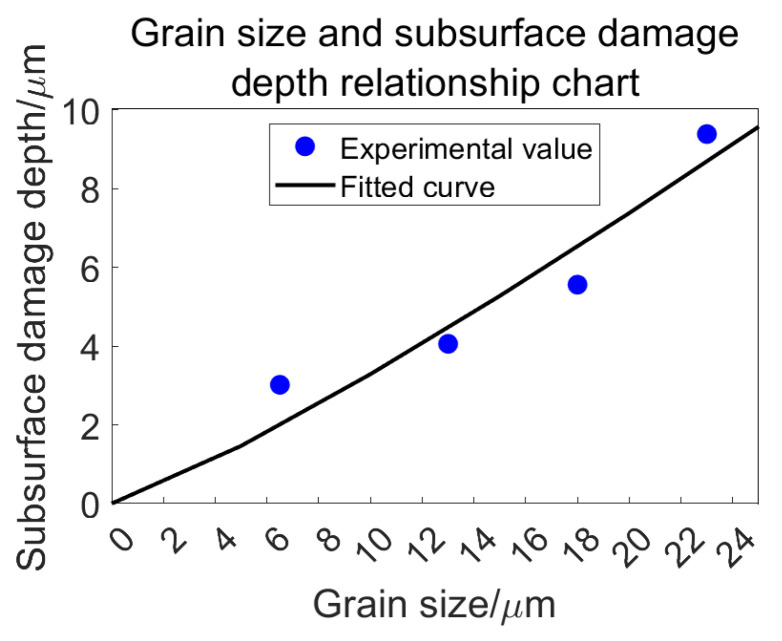
Relationship between abrasive grain size and subsurface damage depth.

The equation demonstrates that the cutting depth of effective abrasive grains is correlated with the depth of radial cracks. By substituting the radial crack depth for subsurface damage depth and referencing Figure 26, it becomes evident that the relationship between grinding pressure and subsurface damage depth is nonlinear.

#### 3.4.2. The Relationship Between Grinding Pressure and Subsurface Damage Depth 

**Figure 27:**
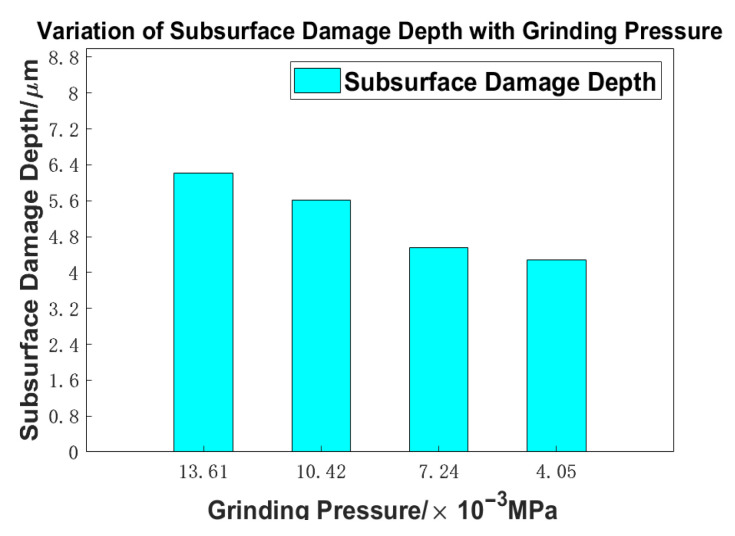
Variation in subsurface damage depth with grinding pressure.

Building upon the analysis presented in the preceding section, we derive the relationship between grinding pressure and subsurface damage depth as follows (Table 9):

A curve can be illustrated as depicted in Figure 27:

From Figure 27, it can be observed that as grinding pressure decreases, the depth of subsurface damage also diminishes. The increase in subsurface damage depth is more pronounced when transitioning from 13.61 × 10^−3^ MP to 7.24 × 10^−3^ MPa, whereas the difference between 7.24 × 10^−3^ MPa and 4.05 × 10^−3^ MPa is comparatively smaller. Under identical conditions, higher pressures result in deeper penetration of abrasive grains into the glass surface, which consequently leads to more severe subsurface damage. In contrast, at lower pressures, the variation in pressure experienced by each grain is reduced, resulting in more uniform material removal effects.

Based on Equation (7), the expression for the radial crack induced by a single abrasive grain can be derived as follows:

(20) $$c_{m}={\alpha_{k}}^{\frac{2}{3}}{\left(\frac{E}{H_{V}}\right)}^{\frac{2\left(1-m\right)}{3}}{\left(\cot\varphi \right)}^{\frac{4}{9}}{\left(\frac{F_{n}}{K_{IC}}\right)}^{\frac{2}{3}}.$$

The grinding of the workpiece by the grinding disc results from the collective action of numerous abrasive grains [24]. Let s represent the number of abrasive grains, which is determined by the characteristics of the grinding disc. Consequently, we can derive the following formula:

(21) $$F_{s}=\sum_{i=1}^{s}F_{ni}.$$

In the formula, *F_ni_* represents the normal force acting on the i-th abrasive grain, and *s* denotes the number of effective abrasive grains present in the grinding disc.

Considering the ongoing wear of abrasive grains during the grinding process, the value is subject to continuous variation. By integrating Equation (21), we can derive the relationship between radial crack depth and grinding pressure as follows:

(22) $$c_{ma}=\int_{\varphi_{1}}^{\varphi_{2}}{\alpha_{k}}^{\frac{2}{3}}{\left(\frac{E}{H_{V}}\right)}^{\frac{2\left(1-m\right)}{3}}{\left(\cot\varphi_{i}\right)}^{\frac{4}{9}}{\left(\frac{\sum_{i=1}^{s}F_{ni}}{K_{IC}}\right)}^{\frac{2}{3}}.$$

In the formula, *c_ma_* is the depth of radial cracks generated during grinding; φ_1_ is the initial sharpness angle of the grinding particles; φ_2_ is the final sharpness angle of the grinding particles; and φ*_I_* is the sharpness angle of the i-th grinding particle.

**Figure 28:**
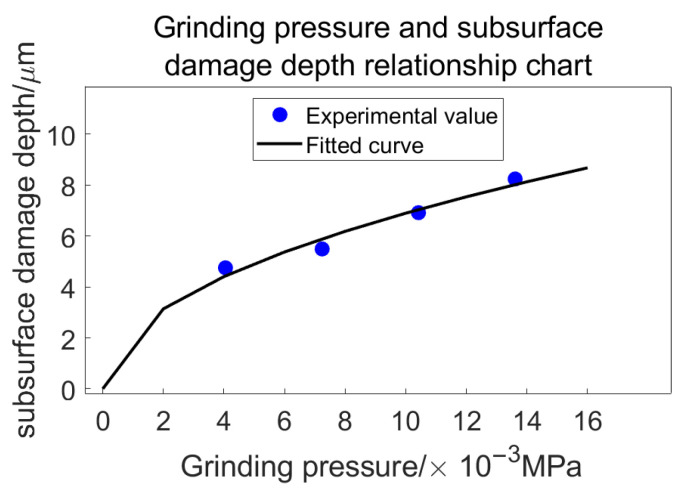
Relationship between grinding pressure and subsurface damage depth.

Since *F_ni_* and φ are subject to constant variation, we can qualitatively assess the relationship between grinding pressure and radial crack depth. By substituting radial crack depth for subsurface damage depth and referring to Figure 28, it becomes evident that the relationship between grinding pressure and subsurface damage depth is nonlinear.

### 3.5. Correlation Between Subsurface Damage Depth and Surface Roughness

Substituting the radial crack depth and the transverse crack depth for the subsurface damage depth (*h_SSD_*) and surface roughness (*R_Z_*), respectively, allows us to derive a relationship between the median crack depth and the transverse crack depth. This can be achieved by combining Equations (7) and (8):

(23) $$\frac{c_{m}}{h}=\frac{{K_{IC}}^{\frac{3}{2}}\cdot {\alpha_{k}}^{\frac{2}{3}}\cdot {F_{n}}^{\frac{1}{6}}\cdot {\left(\cos\varphi \right)}^{\frac{1}{9}}\cdot E^{\frac{2-5m}{3}}\cdot {H_{V}}^{\frac{10m-1}{6}}}{0.43\cdot {\left(\sin\varphi \right)}^{\frac{1}{2}}}.$$

Furthermore, by removing the normal load *F_n_*, the following equation can be derived:

(24) $$\frac{c_{m}}{h^{\frac{4}{3}}}=\frac{3.08\cdot {\alpha_{k}}^{\frac{2}{3}}\cdot E^{\frac{2-6m}{3}}\cdot {H_{V}}^{2m}}{{\left(\sin\varphi \right)}^{\frac{2}{3}}\cdot {K_{IC}}^{\frac{2}{3}}}.$$

Based on Equation (24), the relationship between radial crack depth and transverse crack depth has been established. In this context, *E* represents the elastic modulus (70 GPa for K9 glass); φ denotes the angle of the Vickers indenter tip (ranging from 20° to 80° for grinding particles); *H_V_* indicates the Vickers hardness of K9 glass (6.1 GPa); and *K_IC_* refers to the fracture toughness of K9 glass (0.82 MPa·m^1/2^).

By substituting Equation (6) along with the material parameters into Equation (24), the following expression can be derived:

(25) $$\frac{c_{m}}{h^{\frac{4}{3}}}=59.7136\cdot \frac{{\left(0.09m-0.003\right)}^{\frac{2}{3}}}{{\left(\sin\varphi \right)}^{\frac{2}{3}}}\cdot 0.0871^{2m}.$$

Equation (25) indicates that the angle of the indenter tip, φ, is an independent variable that does not correlate with other parameters. Consequently, the relationship between radial and transverse crack depths can be expressed in terms of m. Therefore, Equation (25) can be reformulated as follows:

(26) $$\frac{c_{m}}{h^{\frac{4}{3}}}=f\left(m\right)=k\cdot {\left(0.09m-0.003\right)}^{\frac{2}{3}}\cdot 0.0871^{2m}.$$

In the equation, *k* is a constant related to φ.

Differentiating Equation (26) results in the following:

(27) $$\left\{\begin{array}{l} {f_{1}}^{\prime }\left(m\right)=0.06\cdot {\left(0.09m-0.003\right)}^{-1/3}\cdot 0.0871^{2m}\\ {f_{2}}^{\prime }\left(m\right)=-4.88\cdot 0.0871^{2m}\cdot {\left(0.09m-0.003\right)}^{2/3}\\ {f_{3}}^{\prime }\left(m\right)=k\cdot \left({f_{1}}^{\prime }\left(m\right)+{f_{2}}^{\prime }\left(m\right)\right) \end{array}\right..$$

The two values derived from the condition where the derivative equals zero are as follows:

(28) $$\left\{\begin{array}{l} m_{1}=1/30\\ m_{2}=0.17 \end{array}\right..$$

Between *m*_1_ and *m*_2_, we have *f′(m)* > 0, indicating that the function is monotonically increasing within this interval. Typically, the value of m lies in the range of 1/3 to 1/2. Consequently, within the interval from 1/3 to 1/2, the function exhibits a monotonically decreasing trend. This implies that *f(m)* attains its maximum value at m = 1/3 and its minimum value at m = 1/2, as illustrated below:

(29) $$\frac{c_{m}}{h^{\frac{4}{3}}}=\left\{\begin{array}{l} \frac{1.056}{{\left(\sin\varphi \right)}^{\frac{2}{3}}}\;\;m=\frac{1}{3}\\ \frac{0.628}{{\left(\sin\varphi \right)}^{\frac{2}{3}}}\;\;m=\frac{1}{2} \end{array}\right..$$

The relationship between radial and transverse crack depths, as illustrated in Equation (29), is contingent upon the variable. Given the ranges from 20° to 80° [25], it follows that Equation (29) remains positive and exhibits a monotonically decreasing trend. The maximum value is attained at φ=20°, while the minimum value occurs at φ=80°. By substituting these values into Equation (29), we derive the following formulas:

(30) $$\frac{c_{m}}{h^{\frac{4}{3}}}=\left\{\begin{array}{l} \frac{1.056}{{\left(\sin20^{{}^{\circ}}\right)}^{\frac{2}{3}}}=2.159\;\;m=\frac{1}{3},\varphi =20^{{}^{\circ}}\\ \frac{0.628}{{\left(\sin80^{{}^{\circ}}\right)}^{\frac{2}{3}}}=0.634\;\;m=\frac{1}{2},\varphi =80^{{}^{\circ}} \end{array}\right.$$

Therefore, the formula establishing the relationship between radial crack depth and transverse crack depth has been derived as follows:

(31) $$c_{m}=\left(0.634\sim 2.159\right)h^{\frac{4}{3}}.$$

Therefore, the formula establishing the relationship between subsurface damage depth and surface roughness has been derived as follows:

(32) $$SSD=\left(0.634\sim 2.159\right)SR^{\frac{4}{3}}.$$

**Figure 29:**
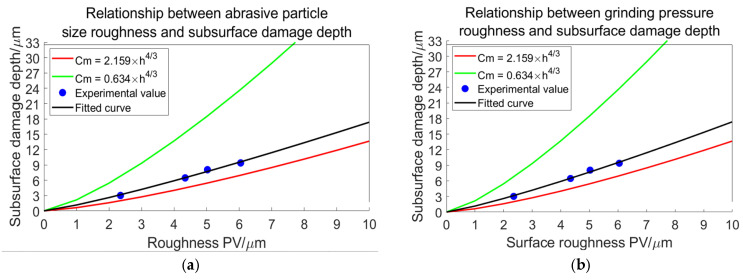
Relationship between roughness and subsurface damage depth. (**a**) Relationship between abrasive particle size roughness and subsurface damage depth. (**b**) Relationship between grinding pressure roughness and subsurface damage depth.

In Figure 29, the trend of the fitting curve illustrating roughness and subsurface damage due to abrasive particle size in (a) exhibits a higher correlation than that observed for grinding pressure in (b) at low levels of roughness. However, as roughness increases, the influence of grinding pressure becomes increasingly significant.

### 3.6. Refinement of Subsurface Damage Detection

A relationship model was established and validated, elucidating the interconnections among abrasive particle size, grinding pressure, surface roughness (PV), and subsurface damage depth (SSD), based on the principles of indentation fracture mechanics.

The influence of abrasive particle size and grinding pressure on the depth of subsurface damage was systematically investigated. Experimental findings demonstrated that the depth of subsurface damage decreased with a reduction in both abrasive particle size and grinding pressure. Employing radial crack depth as an indicator for subsurface damage depth revealed a nonlinear relationship between these parameters and subsurface damage, which was corroborated by the experimental analysis.

A relationship model between surface roughness and subsurface damage was developed based on sharp indenter theory. The model is expressed as SSD = (0.634~2.159)SR^4/3^. The accuracy of the model was validated using experimental data. The fitting curve derived from experimental data lies within the established upper and lower limits, demonstrating alignment with the theoretical model. The relationship between abrasive particle size/grinding pressure and surface/subsurface quality is found to be proportional.

## 4. Conclusions

In addressing the surface and subsurface quality issues during the grinding process of K9 optical glass, this study is based on the principles of indentation fracture mechanics. The experimental variables include two processing parameters: abrasive particle size and grinding pressure. A systematic and in-depth investigation was conducted on the material removal rate, surface quality, and subsurface quality of K9 glass. The main contributions and findings of this paper are as follows:

A mathematical model of planetary grinding with bonded abrasives is established. Simulations of the absolute and relative trajectories of the workpiece relative to the grinding plate are conducted. The effect of different speed ratios between the workpiece and grinding plate on surface uniformity is analyzed, with an experimental speed ratio range of 1:1–1:2. Using the selected process parameters, experiments on rough grinding, fine grinding, rough polishing, and fine polishing of K9 glass are performed. The results show a surface quality with Rq and Ra within 0.35 nm and flatness with a height difference of ≤10 nm. The simulated parameters are experimentally validated and provide standard samples for further grinding experiments.

Grinding time is optimized. Glass samples are ground for 4–24 min using 6.5–23 μm grinding plates. The relationships between grinding time and material removal, removal rate, and surface quality (Ra, Sa, PV) are analyzed. A time range of 6–10 min is set for further experiments.

Grinding experiments are conducted with grain size and pressure as variables. Results show removal efficiency is inversely proportional to grain size and directly proportional to pressure, while surface quality (Ra, Sa, PV) is directly proportional to grain size and inversely proportional to pressure. Grain size has a more significant impact on both efficiency and surface quality, which guides the design of the grinding process.

A model linking surface roughness (SR) to subsurface damage depth (SSD) as SSD = (0.634~2.159)SR^4/3^ is validated experimentally. Research shows that reducing grain size and grinding pressure decreases subsurface damage depth in a nonlinear relationship with SSD.

The grinding process is optimized based on grain size and pressure effects. In rough grinding, use large-grit plates and high pressure. In semi-finish grinding, use medium-grit plates and medium pressure. In finish grinding, use small-grit plates and low pressure. Adjust pressure within the same process to enhance quality. In semi-finish grinding, adjustment.

## Figures and Tables

**Table 1:** Grinding experimental parameters.

Index	Parameter
Base Speed/r·min^−1^	150
Workpiece Disc Speed/r·min^−1^	100
Pressure/N	3
Eccentricity/mm	55

**Table 2:** Relationship between abrasive grain size and material removal.

	Grain Size	28 μm	20 μm	14 μm	10 μm
Removal					
Removal Amount/g		54.4	18.1	15.6	13.9
Removal Rate/μm·min^−1^		4.5687	1.5249	1.3113	1.1689

**Table 3:** Pressure parameter settings.

Sample Parameter	1	2	3	4
Pressure/10^−3^ MPa	4.0500	7.2400	10.4200	13.6100

**Table 4:** Relationship between pressure and surface quality.

	Pressure/10^−3^ MPa	13.61	10.42	7.24	4.05
Index					
Ra/μm		0.2320	0.1850	0.1460	0.1820
Sa/μm		0.1970	0.1700	0.1460	0.1470
PV/μm		5.3740	4.8710	4.5240	3.1870

**Table 5:** Relationship between grinding pressure and removal amount.

	Pressure/10^−3^ MPa	13.61	10.42	7.24	4.05
Removal					
Removal Amount/g		0.0347	0.0295	0.0204	0.0248
Removal Rate/μm·min^−1^		2.9726	2.5217	1.7325	2.1241

**Table 6:** Process Parameter Table.

	Technology	1	2	3	4
Types of Variables					
Abrasive Grain Size/μm		10	14	20	28
Grinding Pressure/10^−3^ MPa		4.0500	7.2400	10.4200	13.6100

**Table 7:** Process parameter table.

	Technology	1	2	3	4
Index					
Grinding disc speed (r/min)		200	150	120	100
Workpiece disc speed (r/min)		100	100	80	60
Pressure (N)		3	2	1	0.8
Particle size (μm)		8.0000	4.0000	2.0000	0.0500
Time (min)		10	20	40	60

**Table 8:** Relationship between abrasive grain size and subsurface damage depth.

Grain Size/μm	23	18	13	6.5
Damage depth/μm	9.3917	8.0639	6.4598	3.0180

**Table 9:** Relationship between grinding pressure and subsurface damage depth.

Grinding Pressure/10^−3^ MPa	13.61	10.42	7.24	4.05
Damage depth/μm/	8.2375	6.915	5.4932	4.7523

## Data Availability

The data used in this study are available for sharing. For researchers with legitimate research needs, the data can be obtained by contacting the corresponding author.
